# Drug Resistance Profiles of *Mycobacterium tuberculosis* Complex and Factors Associated with Drug Resistance in the Northwest and Southwest Regions of Cameroon

**DOI:** 10.1371/journal.pone.0077410

**Published:** 2013-10-16

**Authors:** Henry D. Meriki, Kukwah A. Tufon, Pascal N. Atanga, Irene N. Ane-Anyangwe, Damian N. Anong, Fidelis Cho-Ngwa, Theresa Nkuo-Akenji

**Affiliations:** 1 Department of Microbiology and Parasitology, Faculty of Science, University of Buea, Buea, Southwest Region, Cameroon; 2 Department of Biochemistry and Molecular Biology, Faculty of Science, University of Buea, Buea, Southwest region, Cameroon; 3 Regional Technical Group for the Fight against HIV and AIDS, Regional Delegation of Public Health, Buea, Southwest Region, Cameroon; 4 Clinical Diagnostic Laboratory, Faculty of Science, University of Buea, Buea, Southwest region, Cameroon; Institute of Infectious Diseases and Molecular Medicine, South Africa

## Abstract

**Background:**

Anti-tuberculosis drug resistance continues to be a major obstacle to tuberculosis (TB) control programmes with HIV being a major risk factor in developing TB. We investigated anti-TB drug resistance profiles and the impact of socioeconomic as well as behavioural factors on the prevalence of TB and drug resistance in two regions of Cameroon with such data paucity.

**Methods:**

This was a hospital-based study in which 1706 participants, comprising 1133 females and 573 males consecutively enrolled from selected TB and HIV treatment centres of the Northwest and Southwest regions. Demographic, clinical and self-reported risk behaviours and socioeconomic data were obtained with the consent of participants using questionnaires. Culture and drug resistance testing were performed according to standard procedures.

**Results:**

The prevalence of resistance to at least one anti-TB drug was 27.7% and multi-drug resistance was 5.9%. Smoking, concurrent alcohol consumption and smoking, being on antiretroviral therapy for ≤ 12 months and previous household contact with TB patient were independently associated with tuberculosis prevalence, while only previous tuberculosis infection was associated with drug resistance in a univariate analysis.

**Conclusion:**

The study showed a high prevalence of drug resistance TB in the study population with only previous TB infection associated with drug resistance in a univariate analysis. It also provides evidence in our context, of the role of alcohol and smoking in increasing the risk of developing TB, which is more likely in people living with HIV/AIDS. Therefore, it is important for public health authorities to integrate and intensify alcohol/smoking abstention interventions in TB and HIV control programs in Cameroon.

## Introduction

Efforts to curb TB by the logical application of existing strategies such as early detection and treatment following the direct observed treatment strategy (DOTS), have met with only limited success, slowing the rate of increase but failing to make significant progress toward the goal of tuberculosis elimination [1]. The control of tuberculosis globally has been difficult due to the HIV pandemic, the increase in multidrug resistance (MDR) and extensively drug resistance (XDR) tuberculosis [2]. While the lifetime risk of developing active TB is approximately 5-10% in an immunocompetent person following initial infection it can get up to 50% in HIV/TB co-infected persons [3,4]. According to the WHO report, the incidence of tuberculosis in Cameroon stands 177 cases per 100,000 people [5]. Over the past 20 years, the incidence has fluctuated between 204 in 2004 and 81 in 1990. Cameroon’s HIV prevalence rate among adults between the ages of 15 and 49 years is 4.3% [6]. The Southwest and the Northwest regions with over 1.3 million and 1.8 million inhabitants respectively [7] are among the highest HIV burden regions of the country with prevalence ranging between 5.5% and 6.5% [6]. On the other hand TB annual case notification rates for these regions come close to 124 [8] and 105 [9] cases per 100,000 respectively.

For effective TB control, it is essential to appreciate the complex risk factors and socioeconomic magnitude of the disease at the population level [10]. The increased attention on addressing the social determinants of TB has been induced from both within and beyond the TB control programmes, motivated by the rising number of TB cases and the inequitable distribution throughout the world [11]. In 2010, more cases of TB were diagnosed than ever before in human history, and these cases cluster among disfavoured groups such as the poor, needy and ethnic minorities [11].

Most studies have focused on the effect of HIV on TB, but data on the impact of socioeconomic status (SES) as well as behavioural factors on the prevalence of TB and drug resistance in Cameroon are scarce. The limited available data on drug resistance have been concentrated on three (Centre, Western and Littoral) of the ten regions of Cameroon. Here we report drug resistance from two regions with the highest HIV prevalence and assess the impact of socio-demographic, clinical and behavioural factors on the prevalence of tuberculosis and anti-TB drug resistance.

## Methods

### Study design, site and population description

**Figure 1:**
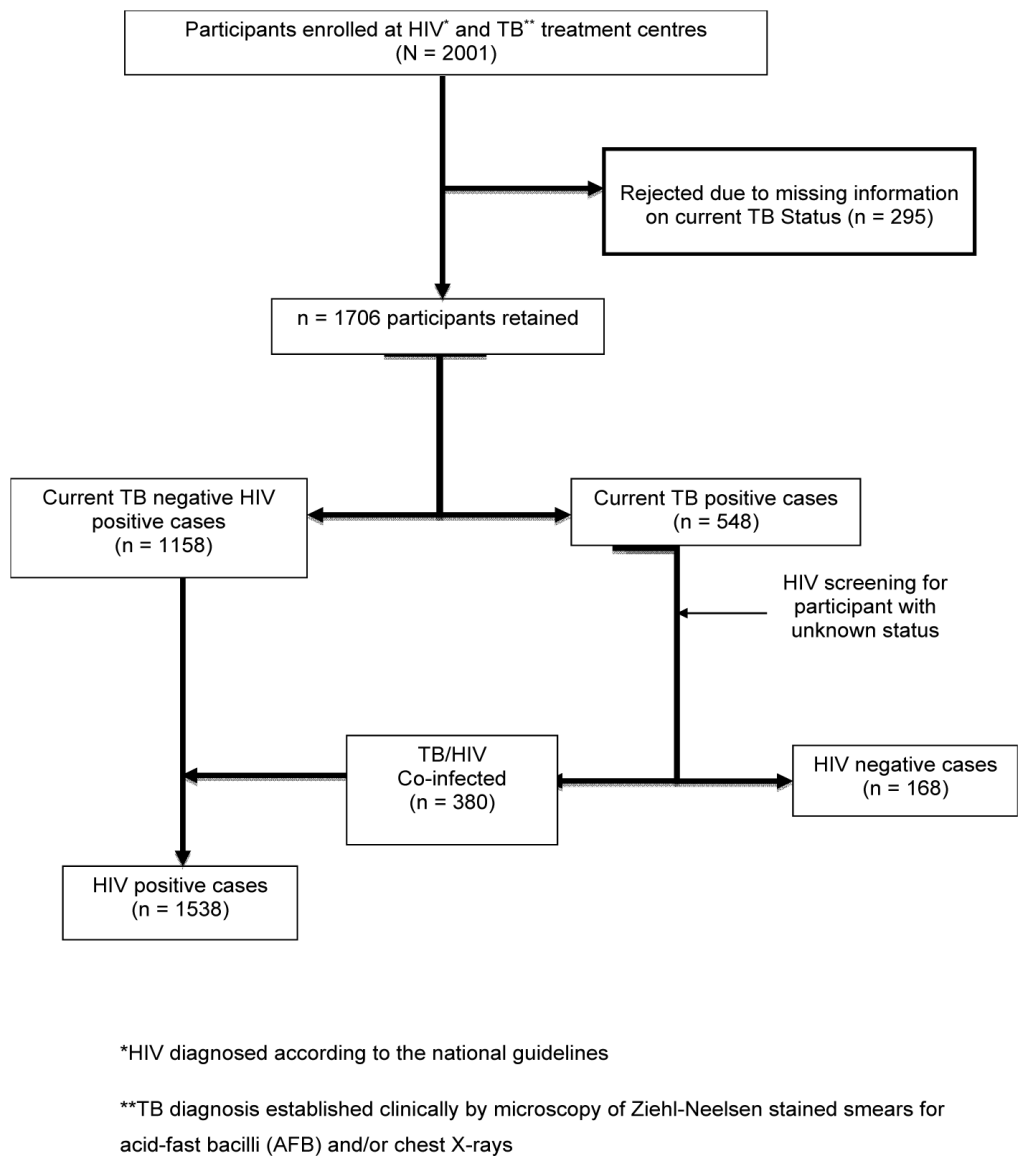
Study population enrollment flow chart.

This was a hospital-based cross-sectional study, carried out between October 2010 and June 2012. Eligible participants (aged ≥18yrs) were recruited for this study from the TB and HIV treatment centre of Bamenda Regional Hospital and St. Theresa Catholic Medical Centre Mambu-Bafut (HIV Management unit only) of the Northwest region; Buea and Limbe Regional Hospitals of the Southwest region. Participants were TB and/or HIV diagnosed cases sent for TB treatment or HIV positive cases qualified for ART or on clinical surveillance. Figure 1 shows the study participants enrolment flow chart.

### Socioeconomic and behavioural variables

A questionnaire was administered to participants and also information extracted from participants’ medical records. Questionaire included information on clinical, socioeconomic and behavioural (active and past smoking and alcohol consumption) data. In the analysis, both active and past smoking were considered as single variable. Likewise both active and past alcohol consumption were considered as single variable. Alcohol consumers were further classified into two categories: standard drinkers and heavy drinkers as defined by clinician guide of the National Institute on Alcohol Abuse and Alcoholism (NIAA) of the National Institute of Health (NIH) [12]. A Standard drink as defined by NIAA is equal to 354.84 ml of beer (approximately 5% alcohol). Men are at risk for alcohol-related problems if their alcohol consumption exceeds 4 standard drinks/occasion and women are at risk if they consume > 3 standard drink/occasion). In Cameroon, the volume of a standard bottle of beer is 650 ml (3 - 8.5% alcohol). Based on NIAA definition, we assumed that standard drinking is 3 bottles/occasion and 2 bottles/occasion of standard Cameroonian beer for men and female respectively.

### Sample collection and processing

Participants presenting with productive cough, one on the spot or early morning sputum specimen was collected using the Precision™ Sputum Collector (Covidien, USA) following standard procedures. Extra-pulmonary samples (lymph node aspirates and pleural fluid) were taken from those initially delivered to the laboratory for diagnosis. These samples were transported in a cold box within 8-24 hours to the Biotechnology unit tuberculosis culture facility, at the Regional Hospital Buea for processing and culture. TB patients without any history of previous anti-tuberculosis treatment were considered as newly diagnosed cases (NDC), while patients with previous anti-tuberculosis treatment or defaulters were considered as previously treated cases (PTC). TB positive participants with unknown HIV status, were counselled, 5 ml of venous blood was collected from them in ethylenediaminetetraacetate tubes and tested following a standard national algorithm. CD4+T-cell count and plasma viral load of HIV positive participants were determined using FACSCount™, (BD Biosciences New Jersey, USA) and the Abbott RealTime HIV-1m2000™ System (Abbott Molecular Inc. Des Plaines, Illinois, USA) respectively following manufacturers’ instructions.

Each sputum sample was decontaminated using N-acetyl-L-cysteine-sodium hydroxide (BD MycoPrep^TM^, Becton Dickinson Diagnostic System, Maryland, USA) following manufacturer’s instructions. For each specimen a slide was prepared for acid fast staining from the sediment after decontamination. Samples were inoculated in triplicate on three Lowenstein-Jensen media slants (one supplemented with 0.4% pyruvate). The cultures were incubated at 37°C and examined weekly for a maximum duration of 8 weeks. Identification was done as previously reported [13]. Briefly, microscopy of Ziehl-Neelsen stained smears, colony morphology, nitrate reduction, niacin accumulation, catalase activity at 25°C and 68°C were used to differentiate members of *Mycobacterium tuberculosis* complex (MTBC).

Drug susceptibility testing was performed on Lowenstein-Jensen medium slants by the standard indirect proportion method of Canetti et al. [14]. Resistance was considered when at least 1% of the number of colonies present on drug-free medium was observed on medium containing 0.2 mg/ml and 0.1 mg/ml of isoniazid (INH), 40 mg/ml of rifampicin (RIF), 2 mg/ml of ethambutol (EMB) and 4 mg/ml of streptomycin (SM). 

Data analysis was carried out using SPSS 18.0 (Statistical Package for the Social Sciences, Chicago, Illinois). Univariate analysis was performed with Chi-square. Logistic regression analysis was used to evaluate significantly associated risk factors in a univariate analysis at a significant level of < 0.05. Odds ratios (OR) and 95% confidence intervals (CI) were presented. 

Ethical approval was obtained from the Cameroon National Ethics Committee (CNEC) (Authorisation N°158/CNE/SE/2010) and administrative approval obtained from the study host institutions and Regional Delegation of Public Health of these regions. All the study participants signed an informed consent form. 

## Results

### Characteristics of the study population

A total of 2001 participants were screened and 1706 were retained; comprising 1133 (66.4%) females (median age: 35 years, interquartile range, IQR: 29 - 43 years) and 573 (33.6%) males (median age: 39 years, IQR: 32 - 46 years) from the North West (696) and South West (1010) regions. The majority (55.9%) of the participants were between the ages of 30-45 years, while 23.1% and 21% of the population were between the 18-29 years and > 45 years old respectively. Of 1706 participants, 67.9% (1158/1706) were HIV positive only, 9.8% (168/1706) were TB positive only, while 22.3% (380/1706) were HIV/TB co-infected patients. Females were predorminantly HIV mono-infected (74.9% [849/1133] females versus 53.9% [309/573] males, p < 0.001), meanwhile males were more HIV/TB co-infected (28.6% [164/573] males versus 19.1% [216/1133] females) and TB mono-infected (17.5% [100/573] males versus 6.0% [68/1133] females). In all, 69.3% of these TB participants were HIV co-infected, while 30.7% were TB mono-infected cases. Most (65%) of the HIV positive paitients were on antiretroviral therapy (ART) with 59.8% having viral load < 1.6log_10_ copies/ml (undetectable viral load) while 40.2% had viral load ≥ 1.6log_10_ copies/ml (median: 4.42log_10_ copies/ml, IQR: 2.94log_10_ -5.30log_10_). The median CD4+ count of HIV patients was 203 cells/µl (IQR: 89-362) (Table 1).

**Table 1:** Demographic and clinical characteristics of the study population.

**Variables**	**N**		**Median [IQR]**				
**CD4** (cells/µl)	839		203 [89 - 362]				
**Viral load** (copies/ml)	1150					**n (%)**	
		Detectable (≥1.6log_10_)	4.42log_10_ [2.94log_10_ - 5.30log_10_]			462 (40.2)	
		Undetectable (< 1.6log_10_)	-			688 (59.8)	
**Variables**				**Current TB status n, (%)**			
**Demographic**	**N**	**Category**	**Prevalence, n (%)**	**Positive**	**Negative**		**χ^2^ p-value**
*Region*	1706	Southwest	1010 (59.2)	84 (8.3)	926 (91.7)		< 0.001
		Northwest	696 (40.8)	464 (66.7)	232 (33.3)		
*Gender*	1706	Male	573 (33.6)	264 (41.1)	309 (53.9)		< 0.001
		Female	1133 (66.4)	284 (25.1)	549 (74.9)		
*Age groups (years)*	1706	18 - 29	394 (23.1)	175 (44.4)	219 (55.6)		0.008
		30 - 45	954 (55.9)	281 (29.5)	673 (70.5)		
		> 45	358 (21.0)	92 (25.7)	266 (74.3)		
**Clinical history**							
*Previous TB infection*	1282	Yes	197 (15.4)	47 (23.9)	150 (76.1)		< 0.001
		No	1085 (84.6)	441 (40.6)	644 (59.4)		
*Household TB contact*	887	Yes	43 (4.8)	17 (39.5)	26 (60.5)		< 0.001
		No	844 (95.2)	67 (7.9)	777 (92.1)		
*Being on ART*	1278	Yes	836 (65.4)	90 (10.8)	746 (89.2)		0.138
		No	442 (34.6)	60 (13.6)	382 (86.4)		
*ART duration*	806	≤ 12 months	229 (28.4)	27 (11.8)	202 (88.2)		0.011
		> 12 months	577 (71.6)	37 (6.4)	540 (93.6)		

ART-antiretroviral therapy, IQR- interquartile range.

Over 40% of the population were currently married (44.2%), and had attended at least a primary school level (46.5%) and over 80% had income levels below 50,000 XAF (Central African CFA franc) (< one hundred US dollar). The prevalence of alcohol consumption (active [47.6%] and past [11.1%]) and smoking (active [15.3%] and past [1.7%]) in the study population was 58.8% and 17% respectively (Table 2). TB was significantly prevalent (p < 0.001) in males (41.1%), in patients without previous TB infection (40.6%), in participants who responded have had previous household exposure (39.5%) (Table 1) and among those who both smoked and consumed alcohol (69%) (Table 2). 

**Table 2:** Socio-economic and behavioural characteristics of the study population.

**Variables**				**Current TB status n, (%)**		
**Socio-economic**	**N**	**Category**	**Prevalence, n (%)**	**Positive**	**Negative**	**χ^2^ p-value**
*Level of education*	1105	Did not go to school	49 (4.4)	01 (2.0)	48 (98.0)	0.044
		Primary	514 (46.5)	50 (9.7)	464 (90.3)	
		Secondary	375 (33.9)	49 (13.1)	326 (86.9)	
		Tertiary	167 (15.2)	13 (7.8)	154 (92.2)	
*Income level*	1105	≤ 50,000 XAF (≤ US$ 100^*^)	932 (84.4)	97 (10.4)	835 (89.6)	0.660
		> 50,000 XAF (> US$ 100)	172 (15.6)	16 (9.3)	156 (90.7)	
*Marital status*	1019	Currently married	451 (44.2)	31 (6.9)	420 (93.1)	0.006
		Previously married	229 (22.5)	04 (1.7)	225 (98.3)	
		Never been married	339 (33.3)	27 (8.0)	312 (92.0)	
**Behavioural**						
*Alcohol*	1455	Yes	855 (58.8)	365 (42.7)	490 (57.3)	< 0.001
		No	600 (41.2)	183 (30.5)	417 (69.5)	
*Alcohol category*	853	Hazardous	386 (45.3)	167 (43.3)	219 (56.7)	0.704
		Standard	467 (54.7)	196 (42.0)	271 (58.0)	
*Smoking*	1455	Yes	247 (17.0)	168 (68.0)	79 (32.0)	< 0.001
		No	1208 (83.0)	380 (31.5)	828 (68.5)	
*Smoking duration*	247	≤ 5yrs	109 (44.1)	84 (77.1)	25 (22.9)	0.007
		> 5yrs	138 (55.9)	84 (60.9)	54 (39.1)	
*Alcoholic-smokers*	870	Smokes and drinks	232 (26.7)	160 (69.0)	72 (31.0)	< 0.001
		Smokes or drinks	638 (73.3)	213 (33.4)	425 (66.6)	

Previously married (widowed and divorced), never been married (single and concubine), XAF-Central African CFA francs,

* 1US$ = ~500 XAF, Standard drink (Female ≤ 2 and males ≤ 3 bottles of beer/occasion), Hazardous drinker (> a standard drink)

### Culture and identification

Of the 548 TB positive participants, 88.35% (484/548) were newly diagnosed cases (NDC) while 11.7% were previously treated cases (PTC). Cultures were positive for 79.7% (437/548) of the samples (52.9% of extra-pulmonary TB [EPTB], 49.1% of smear negative pulmonary TB [SNPTB] and 96.3% of smear positive pulmonary TB [SPPTB]). The remaining 111(20.3%) TB cases although they were culture negative, were treated at the TB treatment centres based on AFB microscopy and/or x-ray radiography.

Biochemical identification analysis was performed in 64.3% (281/437) of isolates with *M. tuberculosis* (64.4%) and *M. africanum* (27.4%) being the major *M. tuberculosis* complex strains (MTBC). Non-tuberculous mycobacteria (NTM) made up 8.1% of the isolates identified. One isolate of the NTM produced a yellowish pigment after exposure to light and was presumptively considered as *M. kansasii*. 

### Prevalence of anti-tuberculosis drug resistance

**Figure 2:**
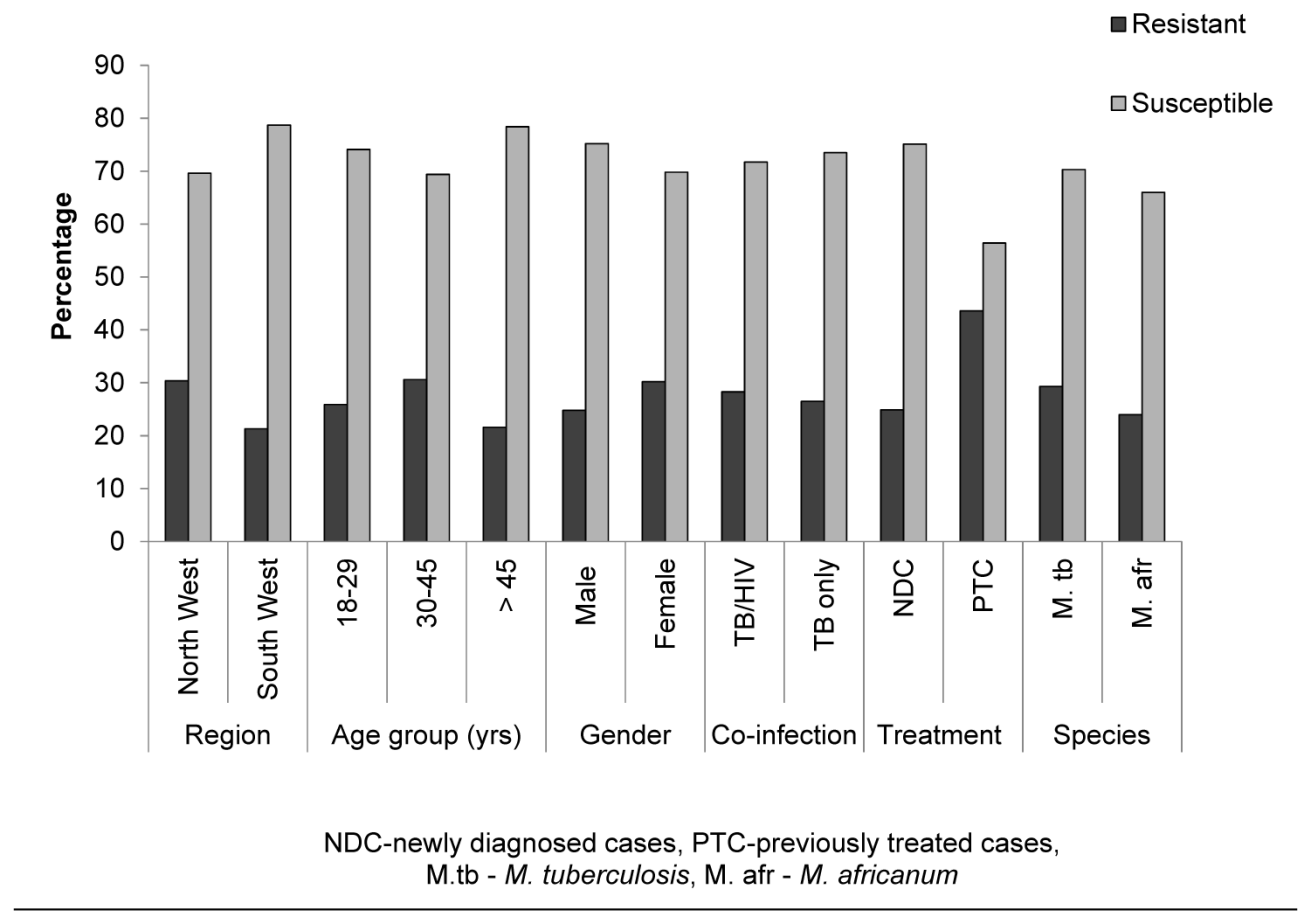
Overall prevalence of drug resistance by region, gender, age groups, *Mycobacterium species*, and TB treatment and co-infection status.

Drug resistance testing was performed in 58.6% (256/437) (identified isolates) of the positive TB cultures. Overall drug resistance to at least a drug was 27.7% (71/256) while 72.3% were susceptible to the entire first-line drugs investigated. Generally, the occurrence of drug resistance was not significantly different (p > 0.05) in the age groups, gender, *Mycobacterium* species, region of residence and among TB co- and mono-infected patients. On the other hand, resistance was significantly prevalent (p = 0.018) among previously treated (41%) than newly diagnosed cases (23%) (Figure 2). 

**Figure 3:**
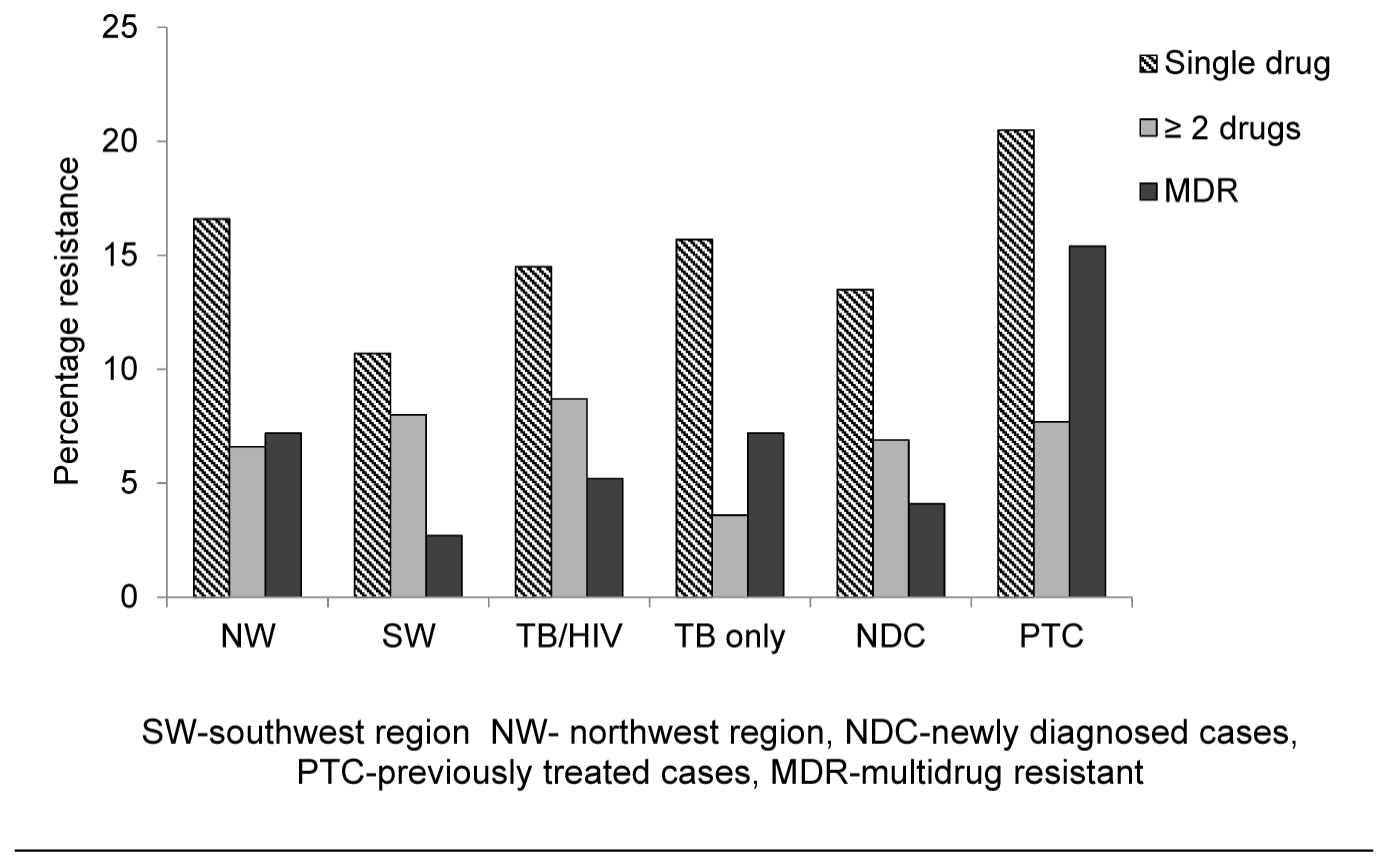
Prevalence of drug resistance patterns.

**Figure 4:**
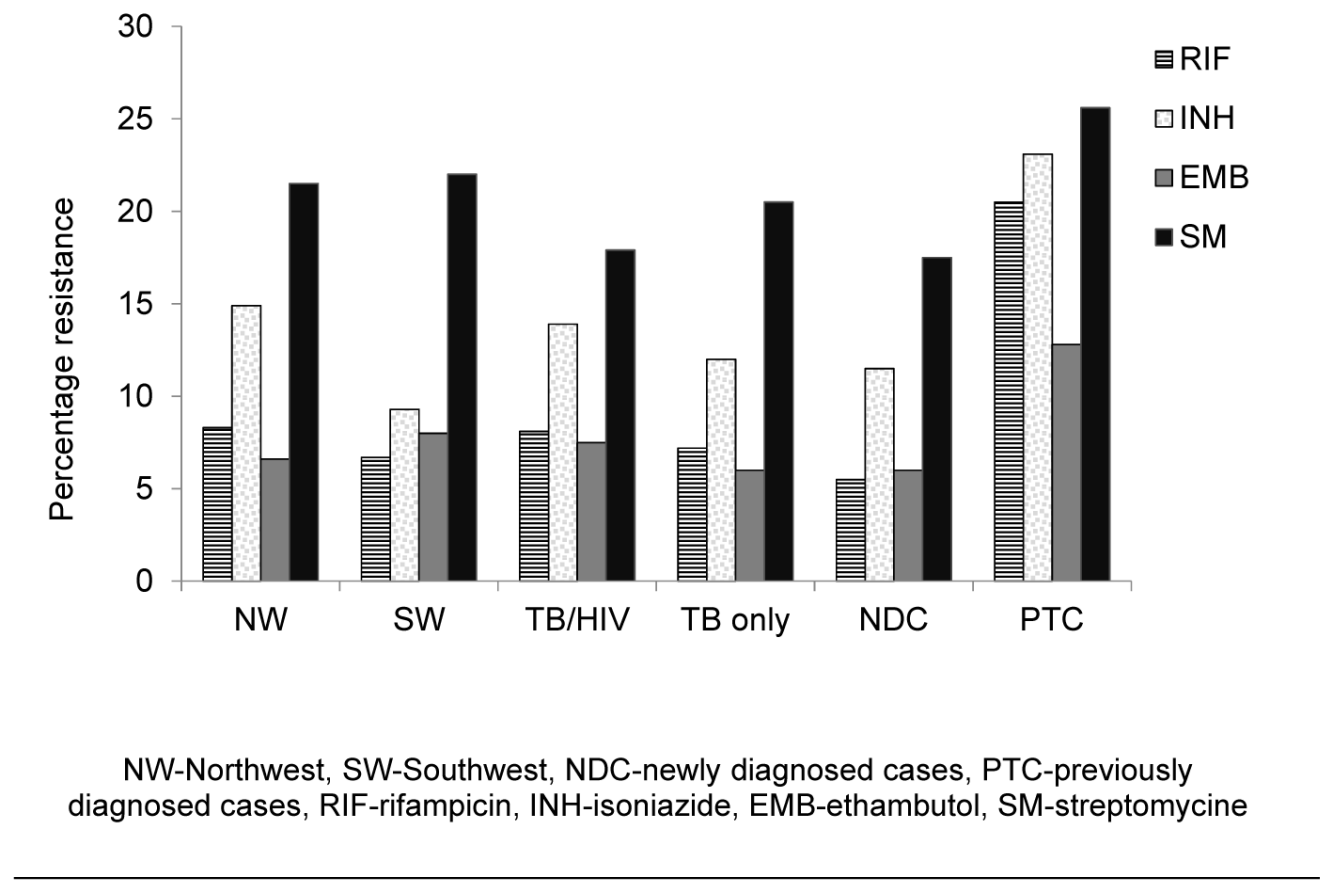
Prevalence of drug resistance to the different first-line anti-TB drugs investigated.

A total of 14.8% (38/256) of isolates were resistant to a single drug (9.7% of SM, 3.9% INH and 1.2% of RIF) and no isolate was resistant to ethambutol only. Resistance to greater than two drugs (excluding MDR) occurred in 7% (18/256) of isolates (Figure 3). MDR was present in 5.9% (15/256) of isolates tested: RIF+INH (three isolates), RIF+INH+SM (nine isolates) and RIF+INH+EMB+SM (four isolates). The prevalence of overall resistance to rifampicin (RIF), isoniazid (INH), ethambutol (EMB), and streptomycin(SM) in the isolates investigated was 7.8%, 13.3%, 7.0% and 18.8% respectively. The prevalence of resistance to each of the drugs was not significantly different (P > 0.05) between TB only and HIV/TB co-infected patients or from those residing in the SW or NW regions. However, resistance to RIF and INH were significantly higher (p = 0.001 and p = 0.005 respectively) in previously treated than newly diagnosed patients (Figure 4).

### Factors associated with the occurrence of TB and anti-TB drug resistance

In a univariate analysis, gender (p < 0.001), age (p < 0.001), history of previous TB infection (p < 0.001), household TB contact (p < 0.001) and being on ART for ≤ 12 months (p = 0.011) showed a significant association with TB occurrence (Table 3). In like manner, there was significant association with alcohol consumption (p < 0.001), smoking (p < 0.001), duration of smoking (p = 0.007) and concurrent smoking and alcohol consumption (p < 0.001) (Table 4). 

**Table 3:** Demographic and clinical factors associated with TB occurrence and drug resistance.

**Factors**		**TB occurrence (95% CI)**				**Drug resistance (95% CI)**	
**Demographic**	**Category**	**Crude odd ratio**	**p-value**	**Adjusted odd ratio**	**p-value**	**Crude odd ratio**	**p-value**
*Gender*	Male	2.55 (2.07-3.16)	< 0.001	0.62 (0.24-1.62)	0.329	0.76 (0.44-1.33)	0.334
	Female	1		1		1	
*Age (years)*	18 - 29	2.31 (1.70-3.15)	< 0.001	1.40 (0.24-8.13)	0.709	1.27 (0.50-3.18)	0.501
	30 - 45	1.21 (0.92-1.59)		0.70 (0.25-1.98)	0.500	1.60 (0.67-3.79)	
	> 45	1		1		1	
**Clinical history**							
*Infection status*	HIV/TB	-	-	-	-	1.10 (0.61-1.98)	0.763
	TB only					1	
*Previous TB infection*	Yes	0.46 (0.32-0.65)	< 0.001	1.37 (0.45 - 4.13)	0.578	2.71 (1.36-5.38)	0.004
	No	1		1		1	
*Household TB contact*	Yes	7.58 (3.92-14.68)	< 0.001	0.20 (0.04 - 0.55)	0.004	1.91 (0.55-6.61)	0.305
	No	1		1		1	
*Being on ART*	Yes	0.77 (0.54-1.09)	0.138	-		0.79 (0.35-1.82)	0.545
	No	1				1	
*ART duration*	≤ 12 months	1.95 (1.16 -3.89)	0.011	0.20 (0.10 - 0.44)	0.001	1.07 (0.31-3.66)	0.912
	> 12 months	1		1		1	

CI-confidence interval, ART-antiretroviral therapy.

**Table 4:** Socio-economic and behavioural factors associated with TB occurrence and drug resistance.

**Factors**		**TB occurrence (95% CI)**				**Drug resistance (95% CI)**	
**Socioeconomic**	**Category**	**Crude odd ratio**	**p- value**	**Adjusted odd ratio**	**p-value**	**Crude odd ratio**	**p-value**
*Level of education*	Low (≤ 7 years of school)	0.77 (0.52-1.14)	0.192	-	-	1.22 (0.51-2.93)	0.658
	High (> 7 years of school)	1				1	
*Income level*	≤ 50,000 XAF (≤ US$ 100^*^)	1.13 (0.65-1.98)	0.660	-	-	1.10 (0.32-3.80)	0.880
	> 50,000 XAF (> US$ 100)	1				1	
*Marital status*	Currently married	1.29 (0.77-2.19)	0.340	-	-	2.11 (0.61-7.30)	0.236
	Never/Previously married	1				1	
**Behavioural**							
*Alcohol*	Yes	1.70 (1.36-2.12)	< 0.001	0.56 (0.19-1.63)	0.288	1.85 (1.0-3.44)	0.051
	No	1		1		1	
*Quantity of alcohol*	Hazardous	1.05 (0.80-1.39)	0.704	-		1.41 (0.73-2.72)	0.305
	Standard	1				1	
*Smoking*	Yes	4.63 (3.46-6.23)	< 0.001	0.20 (0.10-0.52)	0.002	1.15 (0.64-2.05)	0.645
	No	1		1		1	
*Smoking duration*	≤ 5yrs	0.46 (0.26-0.81)	0.007	0.37 (0.04-3.24) ^ᶲ^	0.371	1.61 (0.61-4.21)	0.322
	> 5yrs	1				1	
*Alcoholic-smokers*	Smokes and drinks	4.43 (3.21-6.13)	< 0.001	4.38 (2.31-8.28) ^ᶲ^	< 0.001	0.98 (0.51-1.87)	0.946
	Smokes or drinks	1		1		1	

CI- confidence interval, XAF- Central African CFA Francs, * 1US $ = ~500XAF, Standard drink (Female ≤ 2 and males ≤ 3 bottles of beer/occasion), Hazardous drinker (> a standard drink), ᶲ Model without alcohol and smoking.

Furthermore, when all the above significant variables (except smoking duration, concurrent smoking and alcohol consumption) were included in the model, TB occurrence was once more associated with previous household TB exposure (p = 0.004), being on ART for ≤ 12 months (p = 0.001) (Table 3) and smoking (p = 0.002) (Table 4). When same model was modified by excluding smoking and alcohol consumption while including smoking duration and concurrent smoking and alcohol consumption, only those who both smoked and consumed alcohol were at risk of developing TB (p < 0.001) (Table 4). On the other hand, only previous TB infection was significantly (p = 0.004) associated with drug resistance in a univariate analysis (Table 3).

## Discussion

### Identification and drug resistance profile

Our findings showed that the majority of (88.3%) of the TB study participants were newly diagnosed cases whereas the remainder (11.7%) were previously treated cases. Both the newly diagnosed and re-treatment cases were predominant HIV/TB co-infected patients. With the HIV epidemic driving rapid increases in TB case-notification rates despite implementation of DOTS [15], over half (69.3%) of the study participants were co-infected with HIV. Biochemical identification analysis of isolates revealed that only MTBC strains, predominantly *M. tuberculosis* (64.4%) and *M. africanum* (27.4%) were prevalent supporting previous findings in the West and Centre regions [16,17].

In contrast to a recent report [17], our overall drug resistance to at least a drug was remarkably higher (27.7%) with acquired resistance (AR) (41%) significantly (p = 0.018) higher than initial resistance (IR) (23%). Nevertheless, similar high rates were reported in 1998 [16] and 2000 [18] in the Western and Centre regions of Cameroon. Although not statistically different (p = 0.173), drug resistance was higher in the NW (28.2%) (27.4% IR and 50% AR) than the SW region (20%) (18.3% IR and 33.3% AR). HIV/TB co-infected status, age group, gender, and *Mycobacterium* species did the influence the occurrence of drug resistance (Figure 2). Similar studies carried in Uganda [19], did not find any association between drug resistance, demographic and HIV status.

Generally, resistance to any of the first-line drugs was higher with Streptomycin (18.8%) followed by INH (13.3%) RIF (7.8%) and EMB (7%). This is in agreement with report from other regions of Cameroon [17] although at a much lower rate (2.68% each) and in India at a much higher rate (26.4% and 28.1% for INH and SM respectively) [20]. This study also revealed a high prevalence of RIF and INH resistance among previously treated (RIF, 20.5% versus INH, 23.1%) than newly diagnosed patients (5.5%, RIF versus 11.5%, INH). Other studies have also shown a high resistance to isoniazid and rifampicin in previously treated cases [21]. 

Of the 256 isolates tested, 14.8% (38) were resistant to a single drug (9.7% of SM, 3.9% INH and 1.2% EMB), while resistance to > 2 drugs (excluding MDR) occurred in 7% of these isolates. Multidrug resistance was present in 5.9% of these isolates with significantly (p = 0.021) higher rates among previously treated cases (15.4%) than newly diagnosed cases (4.1%). This was slightly lower (6.67%) than that reported by Assam-Assam et al. [17]. Noeske et al. [22] reported a 51% of resistance to one or more anti-TB drugs and a 12% MDR rate, among smear positive pulmonary TB re-treatment patients in the Littoral region of Cameroon. MDR-TB from other regions of the world ranges from to 12% - 37% in previously treated cases and 2.1% - 12% in newly diagnosed cases respectively in regions of the Americas, Western Pacific, and European regions [5]. Although overall resistance was slightly higher in HIV/TB co-infected (28.3%) than TB mono-infected patients (26.5%), MDR was not significantly different (p = 0.473) among these groups. Contrary, studies carried in the Netherlands during 1993–2001, showed that MDR-TB among newly diagnosed patients was more frequent in those with HIV co-infection than in those with no HIV infection [23]. 

### Factors associated with TB prevalence and drug resistance

Several studies have supported the strong confounding effects of demographic factors such age and gender on the incidence of TB globally [24], [25]. Our results tally with these reports with a high odd ratios in males (OR, 2.55, 95% CI: 2.07 - 3.16) and the age group 18 - 29 years (OR, 2.31, 95% CI: 1.70 - 3.15). However, while females were more infected than males in the age group 18 - 29 years (63.4% females versus 36.4% males, p < 0.001), males dominated in the age group 30 - 45 years (55.5% males versus 44.5% females, p < 0.001). But then, there was no significant difference between males and females in the age group > 45 years (47.8 % males versus 52.2% females, p < 0.097). A higher incidence of TB in females between the ages of 18 - 29 years may be attributed to high prevalence of HIV, a modulatory factor of TB with more females being infected at this early age [26], [27]. 

TB being a disease of poverty plaguing mostly resource-limited countries, the association of speciﬁc socioeconomic factors and TB is unclear [28]. This study did not demonstrate a significant association between TB occurrence or drug resistance and level of income, educational or marital status. However, high level of education and low-income level had more TB cases. Glynn et al. [29] showed that higher socioeconomic status was associated with TB in rural Malawian subjects, probably due to increased awareness and hence greater likelihood of diagnosis. Studies from China also revealed that TB was negatively correlated with per capita income and good household economic conditions were a protective factor [30].

This study showed a high occurrence of TB among individuals without previous history of active TB infection. With over half of our study participants, being co-infected with HIV, an important underlining risk factor [15], may account for this high rate. Household contact studies among TB patients and large epidemiological surveys [31] have established that close contacts of infectious TB cases including household contacts and care givers/health-care workers [32] are at a higher risk of becoming infected with *Mycobacterium tuberculosis* and development of primary active tuberculosis. Consistent with these findings, participants who responded have been in contact with a TB infected house member were more likely to have TB (OR, 7.58, 95% CI, 3.92 - 14.68) than those without. As previously reported, TB can be transmitted within a short period of contact [33] and the opportunities for such interactions are abundant in endemic settings with additional risk such as poverty, overcrowding, and high infection pressure [34]. Casual transmission is therefore a critical factor in TB dynamics in endemic settings [31].

Several studies have demonstrated a direct connection between smoking and TB. In a large-scale meta-analysis in which studies from 14 different countries were assessed, Bates et al. [35] showed that smoking increases one’s risk of latent TB infection by nearly two times, as well as increasing one’s risk of developing TB disease by around two and a half times. Similarly, in a univariate analysis, smoking (p < 0.001) and duration of smoking (p = 0.007) were associated with TB occurrence in our study. Studies have shown that the risk of active tuberculosis is substantially elevated in people who drink more than 40g alcohol per day, and/or have an alcohol use disorder [36]. There was a high prevalence of alcohol consumption among our study participants with about 42% being hazardous alcohol consumption. Alcohol was also associated with TB in a univariate analysis (p < 0.001) and this association was even stronger in those who both smoked and drank alcohol (p < 0.001). According to the NIAAA alcohol alert, about 80 - 90 % of alcoholics, smoke and smokers are 1.32 times more likely to consume alcohol than non-smokers. In this study, 26.7% percent of the study participants both smoked and consumed alcohol. In a population-based case-control study conducted in rural south India, the effects of smoking after adjustment for drinking were more definite than drinking after adjustment for smoking in increasing the incidence of TB [37]. 

Among HIV patients, TB occurrence was not significantly different (p= 0.138) in those who were on ART and those without. Nevertheless, those who have been on ART for ≤ 12 months had higher odd ratio (OR, 1.95, 95% CI: 1.16 - 3.89) of developing TB than those who have been on ART for > 12 months. Suthar et al. [38] showed ART is strongly associated with a reduction in the incidence of tuberculosis irrespective of the CD4 count. Another study by Del Amo et al. [39] showed that the incidence of TB was significantly lower among patients who were receiving ART than among those who were not, but the effect varied across subgroups and according to time since ART initiation. Del Amo et al. [39] further demonstrated that during the first 3 months of ART, patients had an increased risk for TB, particularly if they had a baseline CD4 count <50 cells/mm^3^ or were older than 50 years. Generally, combination antiretroviral therapy significantly reduced the risk of tuberculosis in HIV-infected persons [40].

Multivariate analysis of the significant variables showed smoking, concomitant alcohol consumption and smoking, previous TB infection, previous household contact with TB patient and ART duration were independently associated with TB occurrence. On the other hand, only previous TB infection (p = 0.006) was associated with anti TB-drug resistance in a univariate analysis. This corroborates recent studies by Olusoji and Osman [41]; findings of systematic reviews done in Europe on the risk factors for MDR-TB [42], [43] and in surveys conducted in several countries by the World Health Organization [44]. Both overall TB drug resistance and MDR-TB were not significantly different between HIV/TB co-infected and TB only. Similarly, a systematic review by Suchindran et al. [45] could not demonstrate an association between MDR-TB and HIV or acquired MDR-TB and HIV, but their results suggested that HIV infection was associated with primary MDR-TB. 

## Conclusions

This study showed an overall high prevalence of drug resistance TB in the SW and NW regions. It also demonstrated that smoking, concurrent alcohol consumption and smoking, previous household contact with TB patient, being on ARV treatment for ≤ 12 months were independently associated with occurrence of TB. While only previous TB infection was associated with TB drug resistance in a univariate analysis. It also provided evidence in our context, of the role of alcohol and smoking in increasing the occurrence of TB especially among people living with HIV/AIDS. Therefore, there is a need for public health authorities to integrate and intensify alcohol/smoking abstention interventions in TB and HIV control programs in Cameroon.

### Study Limitations

Recruitment of study population from treatment units excluded TB suspect who might have been culture positive. 
